# An integrated genetic linkage map for white clover (*Trifolium repens* L.) with alignment to *Medicago*

**DOI:** 10.1186/1471-2164-14-388

**Published:** 2013-06-10

**Authors:** Andrew G Griffiths, Brent A Barrett, Deborah Simon, Anar K Khan, Paul Bickerstaff, Craig B Anderson, Benjamin K Franzmayr, Kerry R Hancock, Chris S Jones

**Affiliations:** 1 AgResearch Grasslands Research Centre, Private Bag 11008, Palmerston North, 4442, New Zealand; 2 Pastoral Genomics, ℅ AgResearch Grasslands Research Centre, Private Bag 11008, Palmerston North, 4442, New Zealand; 3 Landcorp Farming Limited, PO Box 5349, Wellington, 6145, New Zealand; 4 AgResearch Invermay Agricultural Centre, Private Bag 50034, Mosgiel, 9053, New Zealand; 5 Infoics Limited, PO Box 83153, Auckland, 0652, New Zealand

**Keywords:** Legume, *Trifolium repens*, Linkage, Microsatellite, SSR, GeneThresher®, Allotetraploid, Homoeologue, *Medicago truncatula*, Synteny

## Abstract

**Background:**

White clover (*Trifolium repens* L.) is a temperate forage legume with an allotetraploid genome (2n=4×=32) estimated at 1093 Mb. Several linkage maps of various sizes, marker sources and completeness are available, however, no integrated map and marker set has explored consistency of linkage analysis among unrelated mapping populations. Such integrative analysis requires tools for homoeologue matching among populations. Development of these tools provides for a consistent framework map of the white clover genome, and facilitates *in silico* alignment with the model forage legume, *Medicago truncatula.*

**Results:**

This is the first report of integration of independent linkage maps in white clover, and adds to the literature on methyl filtered GeneThresher®-derived microsatellite (simple sequence repeat; SSR) markers for linkage mapping. Gene-targeted SSR markers were discovered in a GeneThresher® (TrGT) methyl-filtered database of 364,539 sequences, which yielded 15,647 SSR arrays. Primers were designed for 4,038 arrays and of these, 465 TrGT-SSR markers were used for parental consensus genetic linkage analysis in an F_1_ mapping population (MP2). This was merged with an EST-SSR consensus genetic map of an independent population (MP1), using markers to match homoeologues and develop a multi-population integrated map of the white clover genome. This integrated map (IM) includes 1109 loci based on 804 SSRs over 1274 cM, covering 97% of the genome at a moderate density of one locus per 1.2 cM. Eighteen candidate genes and one morphological marker were also placed on the IM. Despite being derived from disparate populations and marker sources, the component maps and the derived IM had consistent representations of the white clover genome for marker order and genetic length. *In silico* analysis at an *E*-value threshold of 1e^-20^ revealed substantial co-linearity with the *Medicago truncatula* genome, and indicates a translocation between *T. repens* groups 2 and 6 relative to *M. truncatula*.

**Conclusions:**

This integrated genetic linkage analysis provides a consistent and comprehensive linkage analysis of the white clover genome, with alignment to a model forage legume. Associated marker locus information, particularly the homoeologue-specific markers, offers a new resource for forage legume research to enable genetic analysis and improvement of this forage and grassland species.

## Background

Genetic maps and markers are integral to plant improvement strategies being developed and applied in agricultural genomics. These tools enable trait mapping, marker-assisted selection, genetic resource assessment, comparative genetics, and characterisation of genome evolution and organisation [1-4].

White clover (*Trifolium repens* L.) is a temperate perennial forage legume widely used in pastoral systems. The species produces high quality herbage, hosts *Rhizobia* bacteria that transform atmospheric nitrogen into plant available forms, exhibits compatibility and persistence in mixed species pastures, and contributes to soil quality [5,6]. Propagated sexually by seed and asexually by stolons, it is an outcrossing disomic tetraploid (2n=4×=32) with abundant sequence polymorphism and highly heterogeneous populations [7,8]. White clover progenitors are putatively identified as the diploid species *T. occidentale* and *T. pallescens*[9,10]. The white clover genome is moderately compact, estimated at 1093 Mb (1C; [11]), with high sequence similarity in orthologous genic regions within homoeologous pairs [12].

Minor agricultural species, such as white clover, often lag in the development of genomics resources. A range of marker platforms is now available, and the choice among systems is influenced by genome structure, reproductive biology of the species, and consideration of development costs, scale and system efficiency. Targeting marker discovery to specific genome fractions can influence the effectiveness of a marker resource. Markers in low copy number genic regions, such as expressed sequence tag (EST)-derived sources are more likely to be associated with polymorphisms conferring trait effects and are preferred in agricultural plants, however these markers generally exhibit reduced rates of polymorphism [13,14]. Methylation-filtration targets genomic sequence surveys to genic regions, providing gene-rich marker discovery data [15]. As a marker development resource, these sequences share the gene-associated benefits of EST-derived sequence data and the higher polymorphism rate of genomic-derived sequence data. Methyl-filtered sequences are also free from bias created by enriching or screening genomic libraries for specific simple sequence repeat (SSR; microsatellite) motifs, or using expressed sequence data from specific tissues or plant growth stages.

Marker development from targeted sequence can identify polymorphism based on sequence identity (e.g. single nucleotide polymorphism, SNP) or length, such as SSR arrays. In the absence of reference genomes for white clover and progenitors, homoeologous sequence similarity in genic regions hinders development of an efficient SNP discovery process. This is predominantly due to a high proportion of putative SNP markers *in silico* arising from conflation of orthologous sequence within homoeologous pairs [16]. Reference sequence from progenitor species [9,10] partially overcomes this limitation [17], however SNP discovery and utilisation in white clover remains a challenge.

Polymorphisms in candidate gene (CG) sequence offer markers with potential functional effects to enrich maps and advance the genetic dissection of some traits [18]. Although a relatively laborious process, sequencing CGs can be used to identify haplotypes and overcome the limitations of *in silico* SNP discovery experienced in sequence databases [16].

Markers using SSR polymorphism are a co-dominant system that is proven, transportable, amenable to semi-automated assay, moderately cost-effective, and scalable. SSR markers have been used in a number of applications in plant improvement [3], and are estimated to occur at a density of one per 4.7 kb in transcribed regions of the white clover genome [19]. Four independent genetic linkage maps of varying completeness and quality based on *Trifolium* SSR markers have been published in white clover [19-22]. Quantitative and qualitative trait maps have also been developed [23-26], and some effort has been made to identify homoeologue sets based on sequence data from the putative progenitor species *T. occidentale*[17,26]. Large mapped [19] and unmapped [27] sets of white clover SSR markers have been made publicly available to augment three small sets of mapped SSR markers [20,28,29]. In addition, substantial marker and linkage map resources in red clover (*Trifolium pratense*) [30] have been applied in white clover and *T. subterraneum* for comparative mapping [19,22,31]. At present, white clover maps have not been integrated across populations. Marker order is also insufficiently resolved among published maps, and homoeologue-specific markers are not available. As a result, only superficial comparisons among independent mapping populations have occurred, and homoeologue matching and integration has not been achieved. In other species, integration of independent mapping populations has enhanced genetic resolution and provided comprehensive relational locus information for disparate marker and population resources [32]. Furthermore, availability of homoeologue-specific markers to the wider white clover research community would provide a valuable resource for data alignment across populations and research groups.

The Trifolieae forage legume model *Medicago truncatula*, with links to the wider legume phyla community and other agricultural crops [33], is of primary interest in white clover comparative genetics. *In silico* referencing of white clover to *M. truncatula* has identified macrosyntenic relationships maintained between these two species [19,34,35], supported by evidence from mapped comparative markers [22]. This has led to the *Medicago* chromosomal nomenclature replacing the initial *Trifolium* nomenclature of Barrett et al. [20].

The objectives of this research were to: establish an integrated genetic linkage map of the white clover genome based on linkage analysis in two independent F_1_ mapping populations; develop candidate gene-targeted markers for traits of interest as a platform for functional markers and to aid genome alignment with other species; identify homoeologue-specific markers; document a comprehensive set of mapped white clover microsatellite markers; and enrich the *in silico* alignment between *Trifolium repens* and *Medicago truncatula*.

## Results

### SSR marker discovery

White clover end sequence data from 186,890 methyl-filtered genomic DNA clones were assembled to generate a GeneThresher® database (TrGT) containing 364,539 unique sequence segments plus consensus assembly sequences. The mean sequence length was 604 nucleotides. Assembly revealed 84,080 contigs and an estimated non-redundant sequence of 147 Mb, equating to an estimated 30% of the white clover genome’s non-methylated fraction. A sequence homology query of the TIGR Eukaryote Orthologous set indicated TrGT contains 14,372 unique genes.

**Figure 1:**
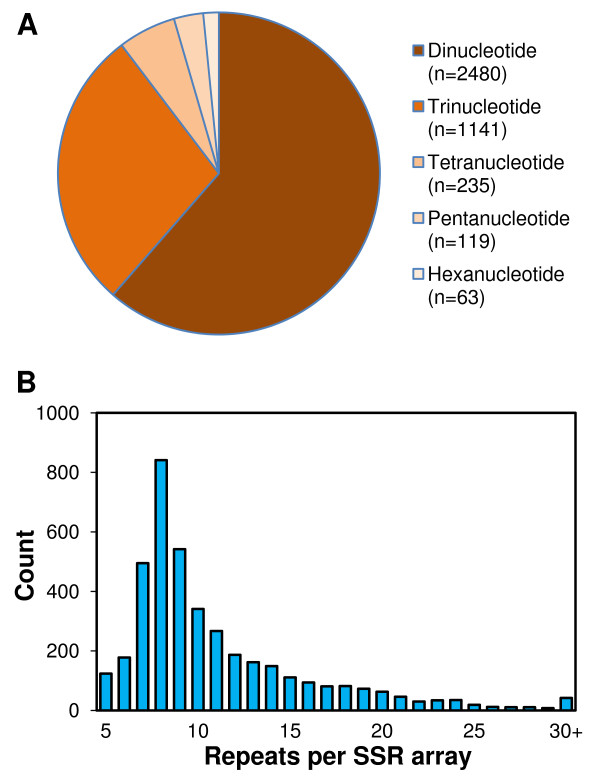
**Sequence repeat characteristics of 4038 SSR arrays mined from methylation-filtered genomic DNA in white clover.** Non-redundant SSRs were identified among 15,647 sequence reads from 186,890 methylation filtered white clover clones of the cultivar ‘Grasslands Huia’, using the GeneThresher® system for methylation filtering. A. Motif type distribution. B. Motif repeat number.

SSR arrays meeting minimum criteria were identified prior to contig assembly in 15,647 singleton sequences in TrGT, which comprise 4.4% of the database sequences. Primer pairs meeting design criteria were identified for 4038 of the arrays including 2480 di-, 1141 tri-, 235 tetra-, 119 penta- and 63 hexanucleotide SSR motif arrays (Figure 1A). The number of SSR repeat units per array ranged from a truncated lower threshold of five up to 55, with a mode of eight (Figure 1B).

Primer pairs for 1344 TrGT-SSRs, including 224 di-, 938 tri-, 108 tetra-, 50 penta- and 24 hexanucleotide SSR motif arrays were synthesised and tested. Of these, 1242 (92%) generated discrete amplicons resolved by capillary electrophoresis following PCR at design parameters. Mean realised amplicon size was 103% of that predicted *in silico*.

### Marker genotyping and linkage analysis

Screening revealed a subset of 465 TrGT-SSRs polymorphic in the parents of population MP2. Their primers and TrGT singleton sequences from which they were derived are disclosed in Additional files 1 and 2, respectively. These SSRs, plus one morphological marker (*Rf*), 18 candidate gene markers (Table 1), 10 ‘ats’ genomic SSRs and 69 ‘prs’ EST-SSRs, identified 733 polymorphic features when genotyped in the MP2 population. This included 268 loci polymorphic in the maternal parent, 201 loci polymorphic in the paternal parent, and 264 loci polymorphic in both parents. The MP2 mapping data were resolved by linkage analysis into sixteen linkage groups in two homoeologous sets and spanned 1265 cM. Linkage groups contained 24 to 70 marker loci and ranged in length from 62 to 95 cM (Additional file 3).

**Table 1:** **Candidate genes mapped in a white clover F**_**1 **_**population**

***T repens *Locus**	**Annotation**	**Intron**	***Tr *LG**	***Mt *PM**	**Ref**	**Forward Primer (5'-3') Reverse Primer (5'-3')**
***TrPHR***	MYB transcription factor	1	2-2	2	[36]	GTTGATGCTGTGGCTCAACTT CCCATAACTCTGAGGACCCCT
	Phosphorus transport					
***TrCPS***	Glutamine synthase	2	3-1	3	[37]	TGGATCAGGTTTGGACTTGAG GGAAGCTGTGAAGGGTCAGTA
	*In planta* nitrogen cycling					
***TrMIP***	Myo inositiol 1-P- synthase	9	3-1	3	[38]	GTTATCTGACCAAGGCTCCTCTG ACCACTGGTGTGCCCGGTGGAAC
	Pinitol synthesis					
***TrLFY***	*LEAFY*	1	3-2	3	[39]	TCTCGATGCTCTCTCTCAAGAAG TCGCTGCACCACCGGCTCCTCCG
	Flowering					
***TrPLT1***	*PLETHORA*	6	4-2	4	[40]	GTGAGCTTCATACCTTCCAGTCC AAGAACTTCGATATATCGCGGTG
	Root growth					
***TrSHP*-*2***	*SHATTERPROOF9*	2	4-2	4	[41]	GAGGTACAAGAAAGCATGTTCAG CTTCAAGTATTCACGGTAACTGC
	Dehiscence					
***TrSHP*-*8***	*SHATTERPROOF9*	9	4-2	4	[41]	AAAGCATGTTCAGATTCTTCTGG GCAGTTTGGCAGCTTCTTGCTGG
	Dehiscence					
***TrPRP***	*ROOT PHOTOTROPISM2*	3	5-2	5	[42]	AATCGACGATGATCTTTACCGCGCCG TTTGTTCTGTGACGCGTGCACACG
	Root growth					
***TrDFR***	Dihydroflavonol reductase (*DFR*)	1	6-1	2	[43]	CATTGGAAGAGTTAGAGATTTAG TCCAACTTCCATAAATGTTCAAC
	Condensed Tannin					
***TrIAA***	*SOLITARY ROOT*	4	6-1	2	[44]	TGAAAGGCTCTGATGCAATTGGG CTAGATTTTTCCATTGCTCTTGG
	Root growth					
***TrSEP1***	*SEPELLATA1*	2	7-1	7	[45]	GCAAACCTGCTAAAGAACTTGAG CTTCAAGTATTCACGGTAACTGC
	Floral initiation					
***TrTT12***	*TRANSPARENT TESTA12*	3	7-1	7	[46]	CAGATCCAAAGTTGGAGACATCGG AGCCCATAGGATATGGTGAAGTGC
	Condensed Tannin					
***TrRMS5***	*RAMOSUS5*	6	7-2	7	[47]	GGCGAAGAAGAAGATGATGG CATCTCTGCATTGAGGTAGCA
	Branching					
***TrAlaAT***	*ACC SYNTHASE1*	6	8-2	8	[48]	AACTGGTGCCTATAGTCATAGTC GCTATTGTATCACGTAAACCCTG
	Flowering					
***TrVP1***	Clover orthologue of *AVP1*	5	8-2	8	[49]	ACTGAATACTATACCAGCAATGC TGCAGGAGTCTGCAACATCTTGC
	Drought tolerance					
***TrPPD***	*PEAPOD*	2	1-1 1-2	no hit	[50]	GATATCGTGAAAAGCGAAAGGAC AGCTTCACTGGAAGGTTCAAATA
	Leaf meristem activity					
***TrANR***	Anthocyanidin reductase (*ANR*)	1	4-2	no hit	[51]	GAGTGCATGTTTGGTTTGCCA TGCAAAGTTCACAGGTGTAGC
	Condensed tannin					
***TrSTP***	*STAMINA PISTILLOIDIA*	n/i	4-1	AC235671	[52]	TGGAAGGTTTTCACCCATCTATG TATCCTGCTGTTCATCCATGGAG
	Flowering					

Eighteen candidate genes related to plant morphology, chemical composition, or transcription regulation were evaluated by *in silico* analysis. Primers were designed from *T*. *repens* genomic sequence, and length polymorphisms were assayed in 92 members of population MP2 using intron-spanning PCR for all loci except *TrANR* and *TrSTP*, which were assayed by SNP and exon size variation, respectively. *Tr* LG=white clover linkage group; *Mt* PM=*Medicago truncatula* genome assembly 3.0 pseudomolecule; Ref=cited reference; no hit=not found in *M*. *truncatula* databases and n/i=no intron in this gene. Note that BAC AC235671 is not incorporated into the *M*. *truncatula* assembly.

The 18 candidate gene markers mapped to 19 discrete loci, with only marker *TrPPD* identifying homoeoloci in group 1. Many of the candidate genes generated additional amplicons which were monomorphic; these may represent homoeoloci and paralogues. Fourteen of the candidate gene markers generated amplicons of the approximate size predicted *in silico* (95-103% of expected size), three were substantially larger (117-140%), and one (*TrSEP1*) was smaller (63%). Markers derived from different introns of the *SHATTERPROOF9* gene (*TrSHP-2* and *TrSHP-8*) mapped to the same locus (Table 1; Additional file 3). The *Rf* locus was polymorphic in parent 20161.21, and mapped to linkage group 8-1.

Although derived from unrelated populations and distinct marker sources, the linkage maps of MP2 (Additional file 3) and MP1 [20] revealed a similar view of the white clover genome in terms of shared marker order and linkage group size (Table 2). Improved map statistics were observed for MP2 (Table 2) and were a reflection of the 49% increase in marker loci and 39% increase in marker density relative to MP1.

**Table 2:** **Summary of two independent white clover F**_**1**_**-derived parental consensus genetic linkage maps and an integrated map**

**Linkage Group**	**No. of Marker Loci**			**Map Length (cM, Kosambi)**						**Marker Density (cM/locus)**		
				**Observed**			**Estimated**					
	**IM**	**MP2**	**MP1**	**IM**	**MP2**	**MP1**	**IM**	**MP2**	**MP1**	**IM**	**MP2**	**MP1**
**1-1**	66	44	31	86.7	84.9	91.0	89.3	88.9	80.8	1.31	1.93	2.94
**1-2**	87	70	27	92.8	93.1	72.0	94.9	95.8	81.8	1.07	1.33	2.67
**2-1**	42	24	22	70.2	63.2	72.0	72.6	72.0	76.8	1.67	2.63	3.27
**2-2**	48	38	18	71.4	70.9	76.0	69.6	77.0	80.8	1.49	1.87	4.22
**3-1**	77	47	35	89.4	92.4	76.0	91.7	96.4	73.8	1.16	1.97	2.17
**3-2**	85	53	38	88.1	87.8	77.0	90.2	91.2	66.8	1.04	1.66	2.03
**4-1**	98	59	52	82.5	76.9	82.0	84.2	79.5	86.8	0.84	1.30	1.58
**4-2**	78	52	47	80.0	76.7	94.0	82.1	79.7	98.8	1.03	1.47	2.00
**5-1**	60	50	26	76.3	62.3	77.0	78.9	64.9	95.8	1.27	1.25	2.96
**5-2**	63	46	14	72.7	65.4	28.0	75.0	68.3	76.8	1.15	1.42	2.00
**6-1**	57	41	22	70.1	68.5	69.0	73.6	68.7	74.8	1.23	1.67	3.14
**6-2**	44	22	23	66.5	70.3	60.0	74.5	74.8	73.8	1.51	3.20	2.61
**7-1**	70	41	38	88.3	94.8	69.0	90.8	99.5	81.8	1.26	2.31	1.82
**7-2**	62	47	20	72.9	76.6	62.0	75.3	79.9	32.8	1.18	1.63	3.10
**8-1**	95	59	41	86.2	93.8	70.0	88.0	97.1	73.8	0.91	1.59	1.71
**8-2**	77	40	39	79.6	87.2	69.0	81.7	91.7	64.8	1.03	2.18	1.77
***mean***	*69*.*3*	*45*.*8*	*30*.*8*	*79*.*6*	*79*.*1*	*71*.*5*	*82*.*0*	*82*.*8*	*76*.*3*	*1*.*20*	*1*.*84*	*2*.*50*
***SEM***	*4*.*3*	*3*.*0*	*2*.*8*	*2*.*1*	*2*.*9*	*3*.*7*	*2*.*0*	*2*.*9*	*3*.*7*	*0*.*06*	*0*.*13*	*0*.*18*
**Total**	**1109**	**733**	**493**	**1273.6**	**1264.8**	**1144.0**	**1312.5**	**1325.2**	**1220.7**			
				**Genome Coverage**			97.0%	95.4%	93.7%			

MP1 is the map published by Barrett et al. [20]; MP2 is based on linkage analysis of 184 individuals from the pair cross of heterozygous genotypes 21125.DC and 20161.21. IM = the linkage map integrated from consensus maps in populations MP1 and MP2. The integrated map consists of 1109 loci including gene-targeted microsatellites, candidate gene markers, and the morphological marker *Rf*. Estimated = estimated maximum map length in each linkage group (=observed linkage group length×[(no. loci+1/(no. loci-1)]; Method 4 of Chakravarti et al. [53]. SEM = standard error of the mean. Genome Coverage = observed map length/estimated map length according to Sekino and Hara [54].

### Multi-population parental consensus map integration

Linkage group matching between populations prior to developing the integrated map (IM) required placement of additional ‘prs’ and ‘gtrs’ markers common to both MP1 and MP2. The aim was to enrich linkage groups to achieve a joining (anchor) locus density <50 cM per joining locus. Numbers of joining markers per group ranged from three (group 3-2) to 15 (group 5-1) with a mean of 7.6 (Table 3). Genotyping of these additional markers in MP1 facilitated resolution of linkage group 5 (formerly G; [20]), the order of which was insufficiently resolved in the original map of this population.

**Table 3:** Map integration data for inter-population homoeologue matching and alignment in white clover

**Linkage Group**	**Single Locus**		**Allele Size**		
	**Match**	**Mismatch**	**Match**	**Mismatch**	**Ambiguous**
**1-1**	3	0	2	0	2
**1-2**	4	0	3	2	2
**2-1**	1	0	1	0	3
**2-2**	2	0	5	0	4
**3-1**	3	0	3	0	1
**3-2**	2	0	1	0	0
**4-1**	2	0	4	0	2
**4-2**	1	0	7	0	2
**5-1**	1	0	7	2	5
**5-2**	0	0	2	0	3
**6-1**	2	0	2	2	3
**6-2**	0	0	2	1	0
**7-1**	2	0	3	1	2
**7-2**	1	0	3	0	4
**8-1**	4	0	2	0	1
**8-2**	2	0	0	0	2

Data are from mapping populations MP1 [20] and MP2, based on analysis of 121 shared marker loci. Markers polymorphic in both MP1 and MP2 were used to match and align homoeologues based on marker loci that were single locus homoeologue-specific (SL-HS). Additional support for homoeologue matching was provided by comparison of allele size of non-single locus shared marker loci. Of the allele size loci, some indicated an alternative matching that was a mismatch to the maximum parsimony allele-size homoeologue alignment, and others did not provide conclusive support for either alignment combination and were designated as ambiguous. In all cases, homoeologue alignment based on shared marker allele size was corroborated by the SL-HS alignment data.

Inter-population homoeologue matches were based primarily on single locus, homoeologue-specific (SL-HS) markers. These markers, identified from SSRs exhibiting single locus segregation patterns in MP2, had been screened further against a panel of 16 individuals including the parents of MP2 and 14 diverse genotypes from cultivars and ecotypes. Markers that amplified a maximum of two alleles per individual across the entire panel were designated SL-HS (Additional file 4) and then genotyped in MP1 to provide homoeologue-specific joining loci. In all cases where multiple independent SL-HS markers mapped in both MP1 and MP2 populations, marker order and relative positions were consistent across populations. There were no instances where multiple SL-HS markers mapped to a single homoeologue in one population then mapped to separate homoeologues in the other population, which would have been classed as a mismatch (Table 3). Presence of multiple SL-HS loci supported matching 10 of the 16 inter-population homoeologue pairs (Table 3). Matching of one inter-population pair of linkage groups to form an IM linkage group identified by default the other inter-population homoeologue match. Multiple SL-HS loci, therefore, identified all inter-population homoeologue groups to be integrated except for IM 5-1 and 5-2, where the match relied on a single SL-HS marker in 5-1 (Table 3).

Additional independent evidence for matched homoeologues between populations was provided by commonality of allele size or allele size range of the mapped joining loci. SSR primer pairs often generated sets of amplicons of contrasting size ranges; those in one size range mapping to a homoeologue, and those in the other size range mapping to the other homoeologue, or elsewhere in the genome, or were uninformative in that population. Allele size, therefore, could be evidence supporting homoeologue matching between populations. Each linkage group from MP2 was aligned with either of the two potential homoeologues in MP1 and assessed for joining locus allele size similarity. There were multiple joining loci per potential inter-population homoeologous pair and not all size-matching loci were consistent in identifying which homoeologues should be aligned. In any pairing, joining loci suggesting an alignment with a homoeologue could be flanked by joining loci suggesting the other homoeologue. Inter-population homoeologue alignment using allele size (Table 3) was, therefore, derived from identifying the homoeologue pairing that maximised the number of joining loci in agreement with the alignment (matches) while minimising the number of joining loci at variance with the alignment (mismatches). In some cases, the allele size ranges overlapped and were therefore not definitive for homoeologue identification. These were classed as ambiguous loci (Table 3).

The allele size-based inter-population homoeologue alignments were made before analysis of the SL-HS markers, and in all cases SL-HS data corroborated the pairings indicated by maximised allele size agreement data. Of the homoeologue alignments, only group 5–1 relied on a single SL-HS locus; however, it was supported by seven allele size matching loci with only two mismatches (Table 3).

**Figure 2:**
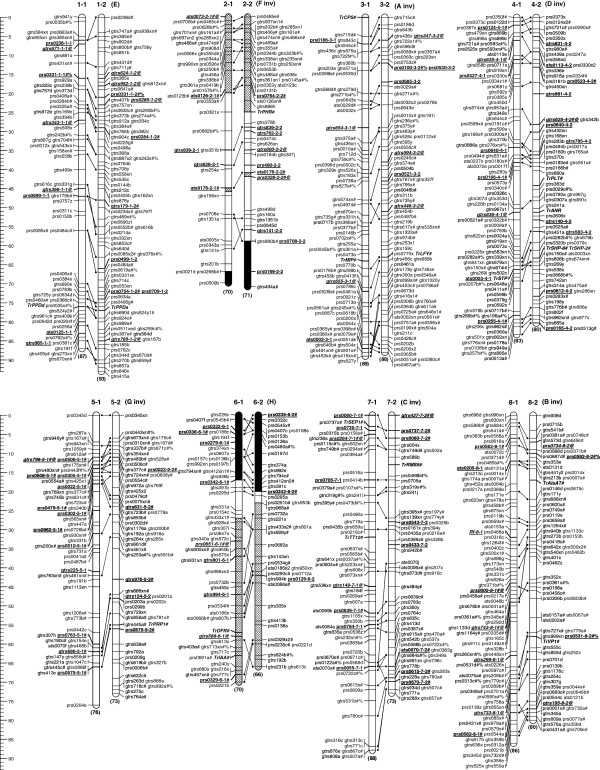
**An integrated linkage map of allotetraploid white clover (2n=4x=32) derived from two independent mapping populations.** The integrated map consists of 1109 independent loci including 427 loci from 308 EST-SSRs, 47 loci from 31 genomic SSRs, 615 loci from 465 GeneThresher®- derived SSRs, 19 loci from 18 candidate genes, and the morphological locus *R*_*f*_. The eight homoeologous pairs of linkage groups have been aligned and orientated with *Medicago truncatula* nomenclature and labelled 1–8. Homoeologues within each pair are designated -1 and -2 based on alignment to homoeologues described in Barrett et al. [20]. For ease of comparison with previous literature, the Barrett et al. [20] A-H nomenclature and relative alignment (inv=inverted) is provided in brackets. Estimated genetic distance (cM) is represented by the scale below the map, and length (cM) of each homoeologue is indicated in brackets below each group. Homoeologous loci are connected by lines between homoeologue pairs. Loci prefixes ats, prs, gtrs and *Tr* denote genomic-, EST-, white clover GeneThresher®-SSRs, and candidate genes, respectively. Loci suffixes a-i, x, xn, y, and z represent locus configurations of individual alleles, (*ab*×*cd*), (*ab*×*cd*) with at least one null allele, (*ab*×*ac*), and (*ab*×*ab*), respectively. Loci labelled in **bold **and ***bold italics@*** denote loci common to both MP1 and MP2 used for map integration, and single locus homoeologue-specific loci for homoeologue identification and integration, respectively. Additional suffixes # and #% represent loci with homology to the *Medicago truncatula* reference genome that align to the equivalent *M*. *truncatula* chromosome, or to a different chromosome, respectively. Regions of homoeologous groups 2 and 6 filled by cross hatching or solid black represent the regions of loci with homology to *M*. *truncatula* chromosomes 2 or 6, respectively.

Upon integration of all matched homoeologues, the resulting IM included 823 molecular and morphological markers identifying 1109 independent loci spanning 1274 cM (Figure 2). The map covered an estimated 97% of the genome, with mean length of 80 cM and 70 marker loci per linkage group (Table 2). Numbers of marker loci per linkage group ranged from 44 to 98, and linkage groups ranged in length from 67 to 93 cM (Table 2). Differences in observed linkage group length within each homoeologous pair ranged from 1% for group 3 up to 21% for group 7. The map was of moderate density, with a mean of 1.2 cM per locus.

Map saturation analysis using mean marker density in the IM estimated that 82% of the genome was within 1 cM of a marker locus, which increased to 100% at 4 cM. This is an increase from MP2 and MP1 at 67% and 55%, respectively, for 1 cM coverage. Substantive gaps of 14 cM on group 1-1 and of 10 cM on group 5-1 were present in each source population and remained after the map integration.

Of the 823 markers in the IM, 204 identified loci on homoeologous groups, including 30% of EST-SSR and 24% of TrGT-SSR primer pairs. A further 43 multi-locus SSRs mapped to non-homoeologous loci. SSR marker loci were generally not clustered by sequence source (Figure 2), suggesting GeneThresher® and EST data are derived from throughout the genome.

There was no evidence of substantive chromosomal rearrangements or re-ordering of marker loci between homoeologues in IM, indicating conservation of homoeologous macrosynteny within this disomic tetraploid (Figure 2). There were, however, minor differences which may be artefacts of linkage analysis or indicative of localised inversions (Figure 2).

### Segregation distortion

**Figure 3:**
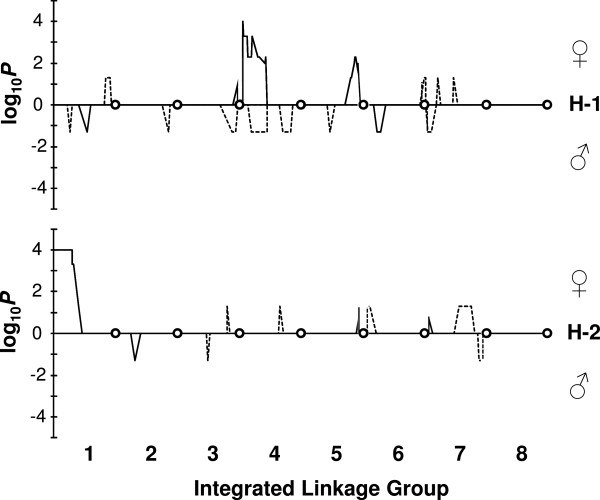
**Segregation distortion patterns in two independent white clover F**_**1 **_**mapping populations.** Data are -log_10_*P* for female (♀)-derived alleles; log_10_*P* value for male (♂)-derived alleles for white clover mapping populations MP1 (dashed line) and MP2 (solid line). Distorted loci were declared at *P*<0.05 (log_10_*P*>±1.3) and aligned as a proportion of distance along each integrated linkage group for each of the two homoeologues (H-1, H-2), noting that -1 and -2 are arbitrary distinctions and do not relate to the progenitor genomes *O* and *P*’ of *T*. *occidentale* and *T*. *pallescens*, respectively. The Self-Incompatibility (*S*) locus [24] is on the top end of group 1, and may underlie the severe male-derived distortion observed in MP2 on that group.

Eight percent of loci in MP1 and MP2 showed segregation distortion, largely restricted to discrete regions of the genome. The specific site of distortion was generally population-specific, except groups 3-1 and 4-1 which showed distortion in both populations (Figure 3). MP2 distortion was derived predominantly from the female parent and was particularly high with a maximum Chi square (*χ*^2^) probability *P-*value threshold of (*P*<0.0001) in groups 1-2, 4-1 and 5-1 (Figure 3). In contrast, segregation ratios in MP1 were less distorted with a maximum *P-*value threshold of *P*<0.05, and were derived in similar proportion from both parents (Figure 3).

### *In silico* genome alignment

**Figure 4:**
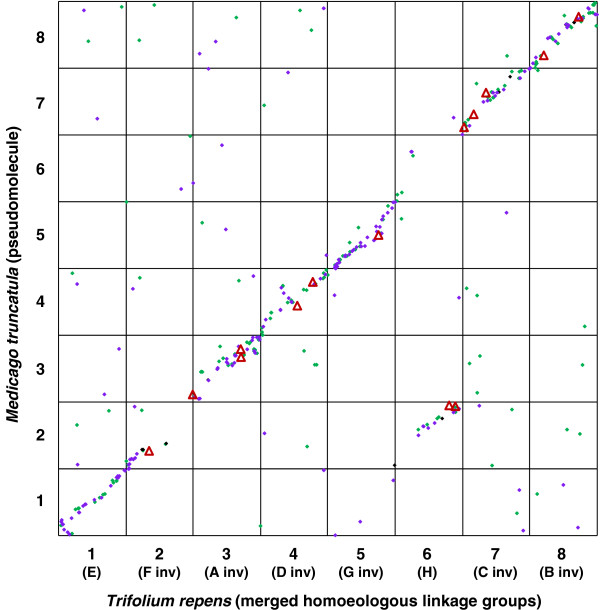
**A matrix plot of synteny assessed between allotetraploid *Trifolium repens *and diploid *Medicago truncatula *genomes.** A total of 376 *T*. *repens* sequences among 822 queried showed sequence homology to *M*. *truncatula*. SSR-containing *T*. *repens* GeneThresher® (purple dots), EST (green dots), and genomic (black dots) sequences and candidate genes (red ∆) were ordered according to proportion of distance along merged *T*. *repens* homoeologue linkage maps (1–8) then aligned by BLAST analysis with homologous (*E*-value threshold of 1e^-20^) sequences in the *Medicago truncatula* genome (v 3.0) which were ordered by proportion of distance along *M*. *truncatula* pseudomolecules (1–8). Candidate genes are in the order of the first 15 listed in Table 1. For ease of comparison with previous *T*. *repens* linkage map literature, the Barrett et al. [20] A-H nomenclature and relative alignment (inv=inverted) is provided in brackets.

The *in silico* alignment of the IM to assembly version 3.0 of the *Medicago truncatula* genome revealed 376 hits at an *E*-value threshold of 1e^-20^ for 822 *T. repens* mapped marker query sequences. Mean alignment span for the 376 hits was 242 bp with a mean *E-*value of 4.4e^-22^. There were similar values for ESTs and TrGT sequences. Inspection revealed 81% of the aligned sequences followed a linear pattern of macrosyntenic alignment with consistent coverage of *M. truncatula* hits across most of the *T. repens* genome (Figure 4). The remaining 19% were more widely scattered (Figure 4). The alignment supports relating the *T. repens* nomenclature of Barrett et al. [20] with *M. truncatula* (Mt) groups as follows: Mt-1 = E with 39 hits; Mt-3 = A with 49 hits; Mt-4 = D with 34 hits; Mt-5 = G with 53 hits; Mt-7 = C with 37 hits; and Mt-8 = B with 50 hits. Groups 3, 4, 5, 7, and 8 as presented in Barrett et al. [20] were inverted in *T. repens* relative to *M. truncatula*; in Figures 2, 4 and 3 and Additional file 3 they have been matched with the *M. truncatula* orientation. Relative to *M. truncatula*, there may be short inversions within white clover groups 1, 4 and 8; however these may be artefacts of constraints in linkage analysis or genome assembly.

*Trifolium repens* groups F and H, in the Barrett et al. [20] nomenclature, revealed a complex relationship with the *M. truncatula* genome. Group F had 22 hits on Mt-2 and three on Mt-6; group H had 16 hits on Mt-2 and six on Mt-6. Moreover, group H had an even distribution of *in silico* hits to *M. truncatula* along the length of the group (Figure 2, Group 6) whereas group F had poor *in silico* alignment to *M. truncatula* with large gaps flanking loci marked by prs328 and ats176 accounting for 47% of group F (Figure 2, Group 2). This was the only *T. repens* linkage group with large regions with no *in silico* alignment to *M. truncatula*. The segment (35%) at the top of group F (Figure 2; 2-1, 2-2) had a dense contiguous alignment spanning the initial 28% of Mt-2 (Figure 4). The bottom segment of group F (18%) contained three hits located in the top segment of Mt-6. Most of group H (66%) (Figure 2; 6-1, 6-2) aligned with and spanned the bottom half of the Mt-2 pseudomolecule (Figures 2 and 4). There was an approximately 5 cM gap between loci prs251 and prs342 (Figure 2) indicating a translocation where the remainder of H (27%) aligned with Mt-6. This alignment comprised three contiguous hits covering the top 14% of Mt-6 then a separate set of three contiguous hits that aligned with the bottom segment of Mt-6 (Figure 4).

## Discussion

In the first linkage map integration across independent mapping populations in white clover, we present a comprehensive analysis of the white clover genome, based on SSR and candidate gene markers aligned to the *Medicago* genome, with a set of mapped molecular markers made available for the research community. This map provides markers enabling homoeologue matching among populations, and thoroughly resolves all linkage groups. Furthermore, the integrated map is a robust composite assessment of the white clover genome, being derived from component linkage maps that reveal very similar data in terms of marker order, genome arrangement and map size; despite being based on dissimilar populations and distinct marker sources. This work complements prior genetic linkage maps [19-22], recent trait-focused studies [24-26], and enriches prior macrosyntenic alignments of *T. repens* with *Medicago*[22,34,35].

### Microsatellite discovery

This integrated map is anchored by gene-targeted SSR markers mined from a white clover GeneThresher® (TrGT) genomic DNA sequence and from ESTs. EST-derived markers exhibit less polymorphism, but have a higher probability of being directly linked to a causative gene than genomic SSRs [3]. Repeat number in EST-SSRs is usually low and a predominance of trinucleotide motifs is explained by changes in other common motif lengths causing frame shifts disrupting coding sequence [3,55]. The white clover EST-SSR source had a preponderance of trinucleotide motifs and a mode of four repeats per array [20], whereas the methyl-filtered TrGT source was predominately dinucleotides motifs, with a mode of eight repeats. Only 71% of EST-derived SSRs produced PCR products [20], compared with 86% [29] and 92% from array targeted white clover genomic libraries [27] and from TrGT-derived SSRs in this study. Intron presence may affect the efficiency of generating amplicons from expressed sequence sourced SSRs, as well as influencing the observed versus predicted amplicon size. Mean observed amplicon size of white clover EST-derived SSRs was 128% of the size predicted *in silico*[20], compared with 103% for TrGT-derived SSRs. Reduced amplification efficiency attributed to M13(-21) primer-based fluorophore addition [56] has been demonstrated [57,58], suggesting that more than 92% of the TrGT-derived SSR primer pairs are viable.

Literature on efficiency of SSR mining from GeneThresher® methyl-filtered sequence is scarce. Gill and co-authors [59] reported 0.8% of sequenced GeneThresher® clones from perennial ryegrass contained SSR arrays. This contrasts with 4.4% of white clover sequences in the present study, and 7% of EST-derived sequences in Barrett et al. [20]. A species-related difference in array density has not been noted in other libraries, and may be a unique feature of the GeneThresher® system interaction with genome size, or other factors. The SSR array density in white clover GeneThresher® and EST sequences are both higher than in a genomic sequence of BAC end surveys [28].

While BLAST results suggest methyl-filtration enriched for genic regions of the white clover genome, 61% of the SSRs were dinucleotide motif repeats. These values agree with genomic DNA surveys in which 48-67% of SSRs found among a range of species are dinucleotides [55]. Inspection of the TrGT database revealed most dinucleotide motif SSRs to be near but outside open reading frames (data not presented) and therefore unlikely to disrupt coding sequences with changes in array length. The increase in SSR array length and polymorphism detected by TrGT-SSRs relative to EST-SSRs also suggests they are from non-coding sequence.

While SSR polymorphism reflects the breeding system and diversity of the subject species, previous studies have shown that genomic SSRs are more informative than EST-SSRs [13,14]. This is supported by the contrast of white clover TrGT-SSRs with the EST-SSR resource of Barrett et al. [20] where a greater proportion of TrGT-SSRs were polymorphic, and more alleles per polymorphic primer were identified.

### Linkage mapping and multi-population map integration

Development of this integrated genetic linkage map relied on parental consensus maps from two unrelated, independent full-sib populations. Furthermore, while these maps were based predominantly on discrete marker sources and MP2 had a greater number of marker loci (49%) and density (39%) relative to MP1, both maps revealed largely similar views of the genome. There was only a 10% increase in map length from 1144 cM to 1264 cM for parental consensus maps of MP1 and MP2, respectively. This indicates most of the recombinogenic genome is mapped and was supported by the high genome coverage calculations, which improved after map integration (Table 2). The IM increases estimated genome coverage to 97%, relative to the prior 95% (MP2) based mainly on GeneThresher®-derived SSRs, 94% (MP1) by Barrett et al. [20] using EST-SSRs and 87% by Zhang et al. [22] which relied primarily on red clover (*T. pratense*) SSRs. Particular features of MP2 and the integrated map are improved resolution of group 5 (formerly G) and extension of homoeologous group 2 (formerly F) as compared to Barrett et al. [20]. Both TrGT and EST marker sets show generalised distribution through the genetic linkage space, indicating both are suitable sources for further marker enrichment of targeted map regions.

Further evidence of the robustness of the assessment of genomic structure provided by these linkage analyses is the consistency of map length and the relative positions of joining loci between the two source maps presented here, as well as unpublished maps developed in our laboratory for both *T. repens* and the diploid progenitor, *T. occidentale*. There are no markers in common between IM and the incomplete genome map of Jones et al. [21], but the trait-focused parental maps of Casey et al. [24] and Wang et al. [26] exhibit regions of general marker order alignment with ‘ats’ and ‘prs’ markers common to IM. Map length is more difficult to compare due to the partial genome coverage of those maps. In contrast, comparative analysis and alignment to the map presented by Zhang et al. [22] of ‘ats’ and ‘prs’ markers in common with IM, indicates significant differences in marker placement both within and among linkage groups. Furthermore, the Zhang et al. [22] map is distinguished by a 47% increase in map length to 1877 cM, relative to the 1274 cM of IM. The recent white clover linkage map [19], based on a combination of white clover, red clover and *Medicago truncatula*-derived SSRs, also exhibits a marked inflation (97%) in total map length to 2511 cM relative to IM. Comparative alignment based on common ‘ats’ and prs’ markers also indicates regions on that map with notable divergence in marker placement relative to IM, particularly linkage groups 2a and 2b [19].

Care was taken in matching homoeologues between the consensus maps of MP1 and MP2 in the map integration, including use of homoeologue-specific SSR markers and allele size matching (Table 3). There is, however, insufficient information in marker and sequence resources to accurately assign linkage groups from this map to progenitor genomes identified by Williams and colleagues [10] and tentatively annotated *O* and *P’*[17]. As additional sequence resources become available, this integrated map and marker resource is expected to accelerate the process of linkage group assignment into homoeologous sets, matching sets to progenitor genomes, and exploration of genome evolution within the genus *Trifolium*.

### Candidate genes

Mapping candidate genes places genes putatively associated with traits of interest on linkage maps. These mapped genes may provide functional markers associated with regions of the genome with a significant effect on trait phenotype, as has been shown in *Medicago*[60], and may be deployed in marker-assisted breeding. Markers derived from two introns of the *SHATTERPROOF9* gene (*TrSHP-2* and *TrSHP-8*) provided an internal control for the intron polymorphism methodology for candidate gene mapping, and mapped to the same locus (Table 1; Additional file 3; Figure 2). While *TrPPD* was the only candidate gene to be mapped in both homoeologues, many of the other genes exhibited additional amplicons that were not informative in the mapping population suggesting they may have loci elsewhere in the genome, including other homoeologues and paralogues. Placement of candidate genes also enables comparative mapping, for example, *LEAFY* (marker *TrLFY*; Table 1) maps to a locus at similar positions in group 3 of our integrated *Trifolium* map and in *Medicago*[60].

### Segregation distortion

Segregation distortion was confined to discrete regions of the genome in both MP1 and MP2, most of which were population-specific (Figure 3) and characterised by flanking markers exhibiting progressive distortion decay with distance from the peak. Zhang et al. [22] also identified discrete regions of segregation distortion, although several individual distorted loci were closely flanked by non-distorted loci without the characteristic distortion decay. In contrast, Isobe et al. [19] documented segregation distortion across much of the white clover genome. It is difficult to accurately align regions of segregation distortion in the parental consensus maps of MP1 and MP2 with the maps of Zhang et al. [22] and Isobe et al. [19] due to discrepancies in marker order where there are SSRs in common. Alignment with the map of Casey et al. [24], in which the white clover *S* locus that regulates self-incompatibility was mapped to the top of a homoeologue of group 1, was straightforward as it contains marker loci in common order. In particular, a single locus homoeologue-specific SSR (prs285) near the *S* locus [24] enables homoeologue matching with MP2, and places the *S* locus at the top of MP2 1-2, which also exhibits strong segregation distortion in the same region (Figure 3). This highlights the value of sharing marker resources to facilitate correspondence of marker and phenotype information across populations, and localises the *S* locus to *T. repens* LG 1-2. MP1 has no segregation distortion on this homoeologue which may be explained by MP1 parents having compatible *S* alleles at this locus. Both MP1 and MP2 share a region of segregation distortion on 4-1 and while white clover is regarded as having a single locus self-incompatibility system [61], the distribution of distortion raises the question of what other loci may influence segregation in these conditions for this species.

### *In Silico* genome alignment

The *in silico* alignment between *T. repens* and *M. truncatula* revealed a general case of co-linearity, and identified an inter-chromosomal rearrangement where Mt-2 and -6 were split across Tr-2 and -6, as first described by Griffiths et al. [35]. Furthermore, orientation of *T. repens* relative to *M. truncatula* was clear in which groups 2 (F), 3 (A), 4 (D), 5 (G), 7 (C), and 8 (B), as oriented in Barrett et al. [20], were inverted relative to *M. truncatula* and reflected that of Griffiths et al. [35]. Groups 1 (E) and 6 (H) were correctly orientated relative to *M. truncatula.* Comparison with *M. truncatula* suggests short inversions compared with white clover groups 1, 4 and 8; however it is not known if these are authentic or are artefacts of constraints in linkage analysis or genome assembly. This is also the first *in silico* alignment of Tr-5 (G), based on the improved marker order and numbers in the integrated map compared with Barrett et al. [20]. Tr-2 (F) was the only *T. repens* linkage group with large regions with no *in silico* alignment to *M. truncatula.* This suggests Tr-2 either has large regions without homology to *M. truncatula*, or regions of *M. truncatula* with homology to actively transcribed regions of the *T. repens* genome have yet to be sequenced. Candidate genes, however, matched expected macrosyntenic sites between *Trifolium* and *Medicago* in all cases, including the group 2/6 translocation as annotated. When considered in totality, this *in silico* comparative analysis confirms a general state of co-linearity between *T. repens* and *M. truncatula*. This extent of alignment suggests the *Medicago* genome can be used as a reference to estimate genome locations of unmapped sequence, and is further supported by evidence of micro co-linearity [12].

While the split of Mt-2 across Tr-2 and −6 was clear, determining which of *T. repens* groups 2 (F) and 6 (H) had greatest co-linearity with Mt-2 was less so. Our data suggest that *T. repens* group H aligns more extensively with Mt-2, although this may only be resolved after development and alignment with a *T. repens* genome assembly. For consistency, however, the published [34] syntenic assignments of Mt-2 = F and Mt-6 = H are maintained. This split of *Medicago* group 2 across *T. repens* groups 2 (F) and 6 (H) is a key feature of the *in silico* alignment. According to a phylogeny of the legume vicioid clade [9], three general groupings, one comprising *Medicago* and *Ononis*, another *Trifolium* and *Melilotus*, and another *Pisum*, *Lathyrus*, and *Vicia*, had diverged from a more ancestral *Cicer arietinum* (Chickpea). Detailed comparative analysis of members of these groupings with *Medicago* shows the group 2 split is a feature of *T. repens*, *Vicia faba*[62], and *Pisum sativum*[63]. In contrast, there is no such split between *Medicago* and *Cicer arietinum*[64], indicating that *Medicago* group 2 may represent the ancestral condition that has since undergone rearrangement during evolution of derived phyla including *Trifolium*.

In contrast to Mt-2 and the other *M. truncatula* pseudomolecules, determining alignment of Mt-6 with *T. repens* was more difficult. This was due to the paucity of matches between Mt-6 and *T. repens*; a total of 10 hits compared to a mean of 52 hits each for other *Medicago* groups aligned with *T. repens*. While Mt-6 has approximately half the sequence data of other *Medicago* groups (http://www.medicago.org/genome/downloads/Mt3/), the very low number of *in silico* matches between Mt-6 and multiple *T. repens* sequence sources is not a surprise for several reasons. Mt-6 is atypical of the other Mt chromosomes as it contains an over-representation of resistance gene analogues and leucine rich repeats [65], the greatest proportion of heterochromatin [66], and a corresponding under-representation of randomly selected and mapped EST markers [65,67]. Furthermore, comparative alignment with other legumes reveals Mt-6 to have reduced marker-based synteny [67]. Since the *T. repens* alignment with *M. truncatula* was based predominantly on exome-derived sequence, reduced synteny with the low gene density Mt-6 is not unexpected and may explain the *in silico* alignment gap identified in Tr-2. The full relationship with Mt-6 may only be resolved after development and alignment with a *T*. *repens* genome assembly.

The *in silico* alignment in this and a previous study [35], used an *E*-value threshold of 1e^-20^ for identifying significant BLASTN matches. Reducing stringency to <1e^-5^ in our analysis revealed numerous spurious matches, often to multiple regions in the *Medicago* genome (data not shown). A similar study by George et al. [34], using a subset of the data from Griffiths et al. [35], derived an *in silico M*. *truncatula*:*T*. *repens* alignment at the <1e^-5^ threshold. While the general patterns of alignment were conserved, the reduced data set and low threshold may have prevented George et al. [34] from determining orientation of *T*. *repens* relative to *M*. *truncatula* for groups F (Mt-2), G (Mt-5), and H (Mt-6). Evidence was also presented for a translocation of a terminal segment of Mt-1 to Mt-3 [34], however there is no evidence for this translocation in the current or previous studies [35], which are augmented significantly by the full EST-SSR dataset, and TrGT-SSRs. Furthermore, there is no evidence in our study of a general breakdown in group 1 synteny as there is a well-supported macrosyntenic relationship along the length of the groups, with a short inversion of Tr-1 relative to Mt-1 at the top end that may be an artefact of linkage analysis. Again, the full relationship between these two species may only be resolved after development and alignment of a *T*. *repens* genome assembly with *Medicago* and other legume genomes.

## Conclusions

This is the first report of integration of independent linkage maps in white clover, and adds to the literature on the utility of methyl filtered GeneThresher®-derived microsatellite markers for linkage map development. A GeneThresher® methyl-filtered gene-targeted SSR marker linkage map (MP2) was generated and merged with an earlier EST-SSR-based consensus genetic map of an independent population (MP1; [20]). Integration required development of homoeologue identifiers to generate the first multi-population integrated map of this disomic tetraploid genome. The integrated map (IM) includes 1109 loci with a total genetic length of 1274 cM, covering an estimated 97% of the genome, and a moderate density of one locus every 1.2 cM. Despite being derived from disparate populations and distinct marker sources, the component maps (MP1 and MP2), and the subsequent IM, provide a consistent and comprehensive view of the white clover genome in terms of marker order and linkage group size. The mapped marker resource, particularly the homoeologue identifiers, provides a vehicle for aligning mapping and quantitative trait loci (QTL) among the forage legume research community, as shown by the ability to align the *S* self-incompatibility locus described by Casey et al. [24] with a region of segregation distortion in MP2. *In silico* comparative analysis at an *E*-value threshold of 1e^-20^ revealed a high degree of co-linearity with the *Medicago truncatula* genome, and a translocation between *T. repens* groups 2 and 6 relative to *M. truncatula*. This provides a platform for comparative mapping and utilising the *M*. *truncatula* genome to assist in clover trait dissection and candidate gene discovery. This work will enable ongoing research in genetic architecture of traits, comparative genetics, genomics, and marker-aided breeding using a mapped marker resource cross-validated in independent mapping populations and aligned to a model forage legume genome.

## Methods

### Plant material

Two white clover (*Trifolium repens* L.) mapping populations were used for the development of the integrated genetic linkage map. Each population was a sample of F_1_ full-sib progeny derived from a hand-pollinated reciprocal pair cross of phenotypically divergent and highly heterozygous genotypes, following a double pseudo-testcross strategy [68]. MP1, the first population (n=92), was used previously to map EST-derived SSRs [20], and was a cross between genotypes ‘6525.5’, a parent of the cultivar ‘Grasslands Sustain’, and ‘364.7’, an individual from a nematode resistance recurrent selection programme [20]. The second population (n=184), designated MP2, was used for linkage analysis to place GeneThresher®-derived genomic SSRs. MP2 was a cross between genotypes ‘20161.21’, a derivative of a bi-parental cross of a genotype from ‘Grasslands Pitau’ and an experimental line, and ‘21125.DC’, a derivative of germplasm sourced in the former Soviet Union. Parent 20161.21 carries the Red Fleck allele at the *Rf* locus [25,69,70], whereas 21125.DC does not exhibit any red flecking. Plants were grown in pots, from which unexpanded trifoliate leaf tissue was harvested. Genomic DNA was purified by an extraction step using the Plant DNAzol system (Invitrogen Corporation, USA) followed by purification and elution from DNA binding columns supplied with the DNeasy extraction kit (Qiagen, USA). Purified genomic DNA was quantitated fluorometrically using Hoechst 33258 DNA-specific dye [71].

### SSR marker discovery

Development of EST-SSR and genomic SSR markers with ‘prs’ and ‘ats’ prefixes, respectively, is previously described [20]. White clover SSRs with ‘gtrs’ prefixes were developed from a *Trifolium repens* GeneThresher® (TrGT) database of genomic DNA sequence enriched for transcriptionally active genome regions using GeneThresher® methylation-filtering technology (Orion Genomics LLC, USA). The TrGT database was developed using DNA from individuals of the white clover cultivar ‘Grasslands Huia’. All TrGT sequences and their contigs were screened for all permutations of di-, tri-, tetra-, penta-, and hexanucleotide SSR repeat motifs using established methods [20]. Screening parameters included exclusion of dinucleotide motif arrays with fewer than six repeats, and fewer than five repeats for the other motif classes.

Primers for SSR arrays were designed from the TrGT singleton database to ensure non-chimaeric source sequence and negate any contig assignment errors. Primer design parameters were 20–27 nucleotides, expected amplicon length 95–395 bp, and Tm of 60°C. Primer design was automated in *Primer3*[72]. Sequences with *phred* scores <50 [73] were excluded. Redundancy reduction using *in silico* PCR and BLAST analysis against the TrGT database prevented retention of multiple primer pairs for a single sequence or legitimate members of the same contig. SSR primer pairs were synthesised (Integrated DNA Technologies, Coralville, IA, USA) with modifications including 5’ M13(-21) tail universal priming site [56] on forward primers, and 5’-GTTTCTT-3’ sequence on reverse primers [74] as described by Barrett et al. [20]. Primers were evaluated for amplification and amplicon size in standard conditions [20].

### Candidate gene markers

Gene orthologues with annotation indicative of interaction with key traits of interest including root morphology, vegetative attributes, flowering and seed production, metabolite pathways, and biotic and abiotic stresses were selected for marker development. Either SSR, intron length, or SNP polymorphism were used to map each candidate gene (CG) marker. Orthologues identified in clover cDNA and TrGT sequence databases were screened for SSR motifs as described. If these motifs were absent or monomorphic, exon-anchored primers were designed to assay for length polymorphism. Intron/exon boundaries were identified by alignment of clover or legume orthologue cDNA with clover genomic sequence orthologues, or publicly available genomic legume databases such as *M*. *truncatula* or *Lotus japonicus* genomic sequence using the EMBOSS (European Molecular Biology Open Software Suite) est2genome programme. Primers were designed with a target product length of 100 to 400bp suitable for resolution by capillary electrophoresis, 5’ and 3’ modified as described by Barrett et al. [20] and synthesised (Integrated DNA Technologies, Coralville, IA, USA).

The anthocyanin reductase (*ANR*) gene sequence contained no SSRs or intron length polymorphisms and was mapped using sequence variation. A region of ~1000 bp spanning Exon1-Intron1-Exon 2 of the *ANR* gene was amplified in MP2 parents, cloned and sequenced using Big-Dye (Version 3.1) chemistry (Applied Biosystems, Foster City, CA, USA) and compared using Alignx (Invitrogen Corporation, Carlsbad, CA, USA). Sequences were sorted into homoeologues based on features specific to the homoeologue most similar to that of *T*. *occidentale*, enabling SNP identification. Primer pairs designed for SNP allele-specific PCR [75] were synthesised (Integrated DNA Technologies, Coralville, IA, USA), products amplified and visualised by ethidium bromide-stained agarose gel electrophoresis and scored for presence/absence.

### SSR, candidate gene and morphological marker genotyping

The incidence of polymorphism in TrGT-SSRs and candidate gene markers was assessed using established assay methods [20] in a white clover genotype panel comprised of MP2 parents and a random sample of six MP2 F_1_ progeny. Informative TrGT-SSR markers were assayed in 184 MP2 F_1_ progeny. To provide loci in common for integration of the MP1 and MP2 parental consensus maps, a set of EST-SSRs mapped in MP1 [20] were assayed in a random subset of 92 individuals from MP2, and a set of TrGT-SSRs mapped in MP2 were assayed in MP1. CG markers were assayed across the subset of 92 MP2 individuals. The morphological marker *Rf* locus was assayed as a presence/absence of the Red Fleck phenotype among 184 MP2 F_1_ progeny.

### Linkage mapping

Genetic linkage analysis and parental consensus map estimation for MP2 used JoinMap® 3.0 software [76] (http://www.kyazma.nl). A loci grouping threshold of LOD≥8 was used with default locus ordering parameters. Estimates of genetic distance were corrected with the Kosambi mapping function. Loci exhibiting significant (*P*<0.05) segregation distortion were retained for initial map estimation, and were removed during locus ordering only if they exhibited distortion patterns incongruous with flanking loci. SSR-derived single parent maps were estimated and merged to form a bi-parental consensus map as described [20] with the nomenclature of George et al. [34]. Final marker order and map estimation was based on multiple ordering iterations and marker combinations until all loci were ordered in the second round analysis. Candidate gene orthologues and EST-derived SSRs were subsequently placed on the TrGT-SSR map. Linkage maps were visualised using MapChart 2.2 software [77].

### Multi-population parental consensus Map integration

Development of a white clover integrated linkage map required matching and alignment of homoeologues between populations. Homoeologue matching was based on multiple lines of evidence, the strongest being placement of single locus homoeologue-specific (SL-HS) SSR markers. Putative SL-HS markers were identified from SSRs with single locus segregation patterns after amplification and mapping in MP2. These markers were further screened against a white clover panel of 16 diverse genotypes including an individual each from cultivars ‘Alice’, ‘Avoca’, ‘Chieftain’, ‘Dacia’, ‘Grasslands Demand’, ‘Grasslands Tahora’, ‘Kotare’, ‘Quest’, and ecotype crosses A1 16/4 (Algeria), A1 28/1 (Algeria), GA 10 (Northern Europe), PxML 6 (Eastern Europe), TR 96–3 (Eastern Europe) and Wab 5 (Western Europe), and the two parents of MP2. SSRs that amplified a maximum of two alleles per individual across the entire panel were designated SL-HS. These SL-HS SSRs were also mapped in MP1 to provide homoeologue-specific joining loci in both MP1 and MP2. Supplementary evidence for inter-population homoeologue matching was based on allele size commonality of joining (anchor) loci placed in both populations at minimum density of <50 cM per joining locus. For some loci, allele size was homoeologue-specific and was used to inform alignment between populations.

Homoeologue match assignment was achieved by sequentially aligning homoeologues from MP1 (eg. 1-1) with the two homoeologue options from MP2 (eg 1-1 and 1-2). Linkage groups of the same homoeologue in different populations were then combined for map integration by matching nomenclature of joining loci and using the 'Combine Groups for Map Integration' function of JoinMap® 3.0 at default parameters [76,78,79].

### Segregation distortion

Linkage map integration and homoeologue alignment enabled comparison of segregation distortion between MP1 and MP2. Probabilities from Chi square (*χ*^2^) tests of segregation ratios against expected ratios of ordered markers were derived [76]. To visualise segregation distortion in MP1 and MP2, *P*-value thresholds (*P*<0.05, <0.01, <0.005, <0.001, <0.0005, <0.0001) for each marker locus were transformed by –log_10_ for alleles derived from the female parent (MP1, 6525.5; MP2, 21125.DC) or log_10_ for alleles derived from male parent (MP1, 364.7; MP2, 20161.21), and aligned with marker position as a proportion of total length for each integrated linkage group.

### Genome length and coverage

The expected genome length based on the parental consensus MP1 and MP2 and the Integrated Map datasets was estimated as described in Method 4 [53], assuming a random distribution of markers. Genome coverage was derived from the expected and observed genome length according to Sekino and Hara [54], and map saturation was calculated as described by Fishman et al. [80].

### *In silico* genome alignment

The integrated white clover genetic linkage map and the *Medicago truncatula* genome sequence were aligned. White clover homoeologue pairs from the integrated map were conflated to form eight homoeologous groups with marker locus positions standardised as a proportion of distance along each linkage group. Sequences harbouring the SSR arrays were trimmed to open reading frame regions of the TrGT data, and to remove 5’ and 3’ untranslated regions from the ESTs. The remaining sequence was then aligned against *M*. *truncatula* Genome Assembly Mt v.3.0 (http://www.medicago.org/genome/downloads/Mt3/) by BLAST analysis with an expect value (*E*-value) exclusion threshold of 1e^-20^ and a maximum of five hits in the *M*. *truncatula* genome. *M*. *truncatula* physical positions were standardised as the proportion of distance along the pseudomolecule for each hit. The strongest hit was retained and plotted against the normalised white clover genetic linkage map locations. Candidate genes mapped in white clover were assessed individually by BLAST for orthologue location in the *M*. *truncatula* assembly.

## Competing interests

The authors declare they have no competing interests.

## Author’ contributions

AGG led the research project including experimental design, data analysis, and manuscript development. BAB contributed to analysis and manuscript preparation; DS and AKK conducted *in silico* analyses. PB organised and queried the GeneThresher® database for SSRs. CBA and BKF conducted the genetic marker data analysis. KRH identified and designed primers for candidate genes and performed *in silico* analyses. CSJ managed the clover genomics research programme and contributed to the manuscript. All authors read and approved the final manuscript.

## Supplementary Material

Additional file 1 **Tabulated white clover GeneThresher®-derived ‘gtrs’ (n=465) and genomic ‘ats’ (n=31) SSR marker and locus summary including primers and sequence identifiers.** SSR name refers to the marker and single locus homeologue-specific markers are denoted with an @ suffix; LG = Linkage group; cM = genetic distance along on linkage group in centimorgans; Type refers to repeat motif size – di=dinucleotide, tri = trinucleotide, tetra = tetranucleotide, penta = pentanucleotide, hexa = hexanucleotide; Motif = repeat motif; Repeats = number of times the motif was repeated in the source SSR array; eSize (bp) = predicted amplicon size (base pairs) based on *in silico* data; Forward and Reverse primers identify the primers flanking the SSR used for PCR amplification; and GeneThresher® Sequence Identifier refers to the unique code of the GeneThresher® sequence (Additional file 2) harbouring the mapped SSR. Click here for file

Additional file 2 **GeneThresher® sequences in FASTA format harbouring mapped SSRs.** GeneThresher® sequence identifiers correspond with those detailed in Additional file 1. Click here for file

Additional file 3 **A white clover genetic linkage map of F**_**1 **_**population MP2 (21125.DC×20161.21).** The linkage map of MP2 contains 733 independent loci including 87 loci from 69 EST-SSRs, 16 loci from 10 genomic SSRs, 608 loci from 465 white clover GeneThresher®-derived SSRs, 21 loci from 19 candidate gene markers, and the morphological locus *R*_*f*_. The eight homoeologous pairs of linkage groups have been aligned and orientated with *Medicago truncatula* and labelled 1–8, and homoeologues within each pair are designated -1 and -2 based on alignment to homoeologues described in Barrett et al. [20]. For ease of comparison with previous literature, the Barrett et al. [20] A-H nomenclature and relative alignment (inv = inverted) is provided in brackets. Genetic length (cM) is represented by the scale below the map, and length (cM) of each homoeologue is indicated in brackets below each group. Homoeologous loci are connected by lines between the two homoeologues. Loci prefixes ats, prs, gtrs and *Tr* denote genomic-, EST-, white clover GeneThresher®-SSRs, and candidate genes, respectively. Loci suffixes a-i, x, xn, y, and z represent locus alleles, (*ab*×*cd*) loci, (*ab*×*cd*) loci with at least one null allele, (*ab*×*ac*) loci, and (*ab*×*ab*) loci, respectively. Loci in **bold** and ***bold italics@*** denote loci common to both MP1 and MP2 used for map integration, and single locus homoeologue-specific loci for homoeologue identification and integration, respectively. Additional suffixes # and #% represent loci with homology to the *Medicago truncatula* reference genome that either align to the equivalent *M*. *truncatula* chromosome, or to a different chromosome, respectively. Regions of homoeologous groups 2 and 6 filled by cross hatching or solid black represent the regions of loci with homology to *M*. *truncatula* chromosomes 2 and 6, respectively. Click here for file

Additional file 4 **Tabulated single locus homoeologue-specific SSRs in white clover.** SSR name refers to the marker; LG = Linkage group; cM = genetic distance along on linkage group in centimorgans; Type refers to repeat motif size – di=dinucleotide, tri = trinucleotide, tetra = tetranucleotide, penta = pentanucleotide, hexa = hexanucleotide; Motif = repeat motif; Repeats = number of times the motif was repeated in the source SSR array; eSize (bp) = predicted amplicon size (base pairs) based on *in silico* data; Forward and Reverse primers identify the primers flanking the SSR used for PCR amplification. Click here for file
