# Supplementary figures and images for: An integrated genetic linkage map for white clover (Trifolium repens L.) with alignment to Medicago
Source: BMC Genomics. 2013 Jun 10;14:388. doi: 10.1186/1471-2164-14-388 (PMC3693905; doi:10.1186/1471-2164-14-388)

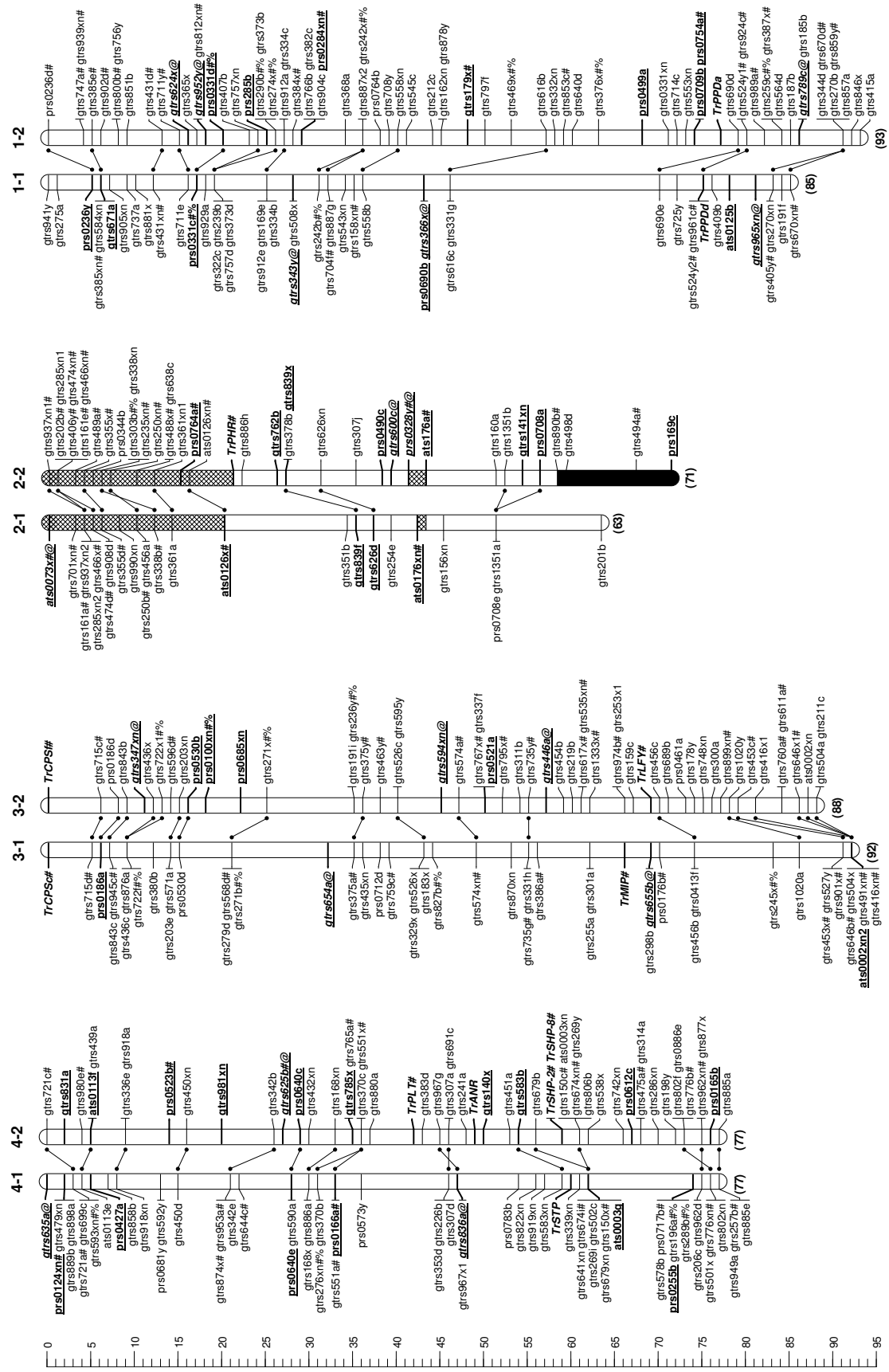

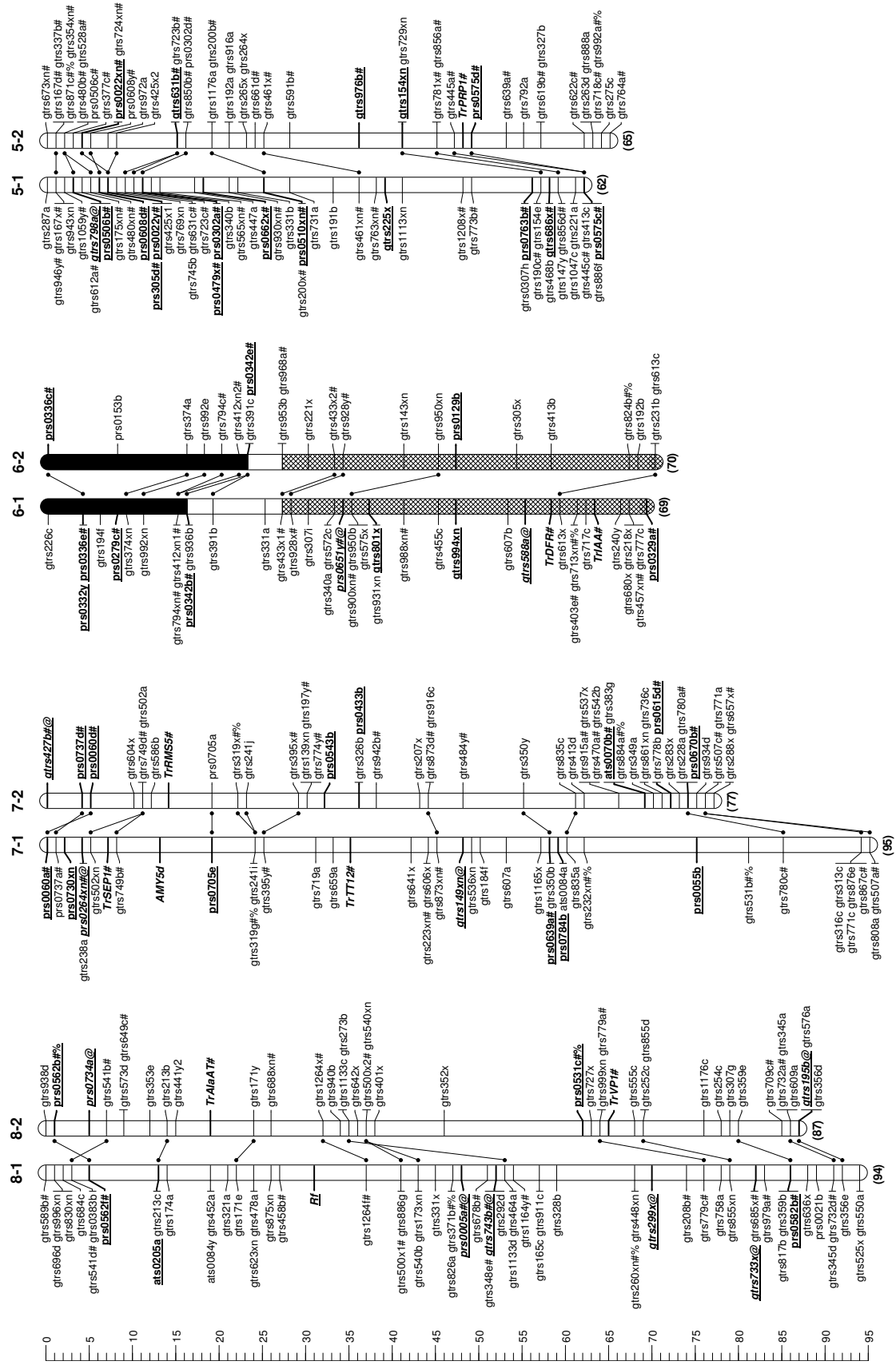

Supplement: Additional file 3 — A white clover genetic linkage map of F1 population MP2 (21125.DC×20161.21). The linkage map of MP2 contains 733 independent loci including 87 loci from 69 EST-SSRs, 16 loci from 10 genomic SSRs, 608 loci from 465 white clover GeneThresher®-derived SSRs, 21 loci from 19 candidate gene markers, and the morphological locus Rf. The eight homoeologous pairs of linkage groups have been aligned and orientated with Medicago truncatula and labelled 1–8, and homoeologues within each pair are designated -1 and -2 based on alignment to homoeologues described in Barrett et al. [20]. For ease of comparison with previous literature, the Barrett et al. [20] A-H nomenclature and relative alignment (inv = inverted) is provided in brackets. Genetic length (cM) is represented by the scale below the map, and length (cM) of each homoeologue is indicated in brackets below each group. Homoeologous loci are connected by lines between the two homoeologues. Loci prefixes ats, prs, gtrs and Tr denote genomic-, EST-, white clover GeneThresher®-SSRs, and candidate genes, respectively. Loci suffixes a-i, x, xn, y, and z represent locus alleles, (ab×cd) loci, (ab×cd) loci with at least one null allele, (ab×ac) loci, and (ab×ab) loci, respectively. Loci in bold and bold italics@ denote loci common to both MP1 and MP2 used for map integration, and single locus homoeologue-specific loci for homoeologue identification and integration, respectively. Additional suffixes # and #% represent loci with homology to the Medicago truncatula reference genome that either align to the equivalent M. truncatula chromosome, or to a different chromosome, respectively. Regions of homoeologous groups 2 and 6 filled by cross hatching or solid black represent the regions of loci with homology to M. truncatula chromosomes 2 and 6, respectively. [file 1471-2164-14-388-S3.pdf]
